# Children's very low food security is associated with increased dietary intakes in energy, fat, and added sugar among Mexican-origin children (6-11 y) in Texas border *Colonias*

**DOI:** 10.1186/1471-2431-12-16

**Published:** 2012-02-20

**Authors:** Joseph R Sharkey, Courtney Nalty, Cassandra M Johnson, Wesley R Dean

**Affiliations:** 1 Program for Research in Nutrition and Health Disparities, School of Rural Public Health, Texas A&M Health Science Center, MS 1266, College Station, TX 77843-1266, USA; 2 UNC Center for Health Promotion and Disease Prevention and Department of Nutrition, UNC Gillings School of Global Public Health, CB # 7461, Chapel Hill, NC 27599-7461, USA

## Abstract

**Background:**

Food insecurity among Mexican-origin and Hispanic households is a critical nutritional health issue of national importance. At the same time, nutrition-related health conditions, such as obesity and type 2 diabetes, are increasing in Mexican-origin youth. Risk factors for obesity and type 2 diabetes are more common in Mexican-origin children and include increased intakes of energy-dense and nutrient-poor foods. This study assessed the relationship between children's experience of food insecurity and nutrient intake from food and beverages among Mexican-origin children (age 6-11 y) who resided in Texas border *colonias*.

**Methods:**

Baseline data from 50 Mexican-origin children were collected in the home by trained *promotora*-researchers. All survey (demographics and nine-item child food security measure) and 24-hour dietary recall data were collected in Spanish. Dietary data were collected in person on three occasions using a multiple-pass approach; nutrient intakes were calculated with NDS-R software. Separate multiple regression models were individually fitted for total energy, protein, dietary fiber, calcium, vitamin D, potassium, sodium, Vitamin C, and percentage of calories from fat and added sugars.

**Results:**

Thirty-two children (64%) reported low or very low food security. Few children met the recommendations for calcium, dietary fiber, and sodium; and none for potassium or vitamin D. Weekend intake was lower than weekday for calcium, vitamin D, potassium, and vitamin C; and higher for percent of calories from fat. Three-day average dietary intakes of total calories, protein, and percent of calories from added sugars increased with declining food security status. Very low food security was associated with greater intakes of total energy, calcium, and percentage of calories from fat and added sugar.

**Conclusions:**

This paper not only emphasizes the alarming rates of food insecurity for this Hispanic subgroup, but describes the associations for food insecurity and diet among this sample of Mexican-origin children. Child-reported food insecurity situations could serve as a screen for nutrition problems in children. Further, the National School Lunch and School Breakfast Programs, which play a major beneficial role in children's weekday intakes, may not be enough to keep pace with the nutritional needs of low and very low food secure Mexican-origin children.

## Background

The Southwestern United States border region is home to many *colonias*. These settlements are occupied by a growing population of people who share a similar Mexican heritage, language, and socioeconomic standing and who have unacceptably high rates of poverty, adult and childhood obesity, and food insecurity [1-3]. Border-region *colonias *can be considered an archetype for the increasing number of new destination immigrant communities [1]. Many of these communities of Mexican immigrants are located throughout the United States, including many non-traditional interior locales [4-6].

Food insecurity underpins an emerging national issue of nutritional health inequity among Mexican-origin and Hispanic households. The 2009 Current Population Survey Food Security Supplement identified low or very low food security in 26.9% of Hispanic households, compared with 14.7% in all U.S. households, and in 18.7% of Hispanic households with food-insecure children compared with 10.6% in all households [7]. In a study of Mexican immigrant families in Minnesota, Kersey and colleagues observed much higher rates of child hunger among 1,310 Mexican immigrant families than among 1,805 non-immigrant families (6.8% versus 0.5%) [8]. Food insecurity is much more prevalent among Mexican-origin households in the Texas border region compared with other regions of the U.S [3,7]. In a study of food access among 610 adult women in Texas border *colonias*, researchers found 49% of all households and 61.8% of households with children could be classified at the most severe level of food insecurity - child insecure [3].

At the same time, nutrition-related health conditions, such as obesity and type 2 diabetes, are increasing in Mexican-origin youth. Risk factors for obesity and type 2 diabetes are more common in Mexican-origin children than other racial/ethnic groups [9-11] and include increased intakes of energy-dense and nutrient-poor foods, such as fats, sweeteners, desserts, and salty snacks. Energy-dense foods are highly palatable and promote higher calorie intakes [12,13]. Diets with proportionally more contribution from energy-dense foods increase the risk for inadequate intakes of vitamin D, calcium, potassium, and dietary fiber and the likelihood of consuming excessive amounts of added sugar, fats, and sodium [14]. For limited-resource populations (households with limited economic and physical resources and limited access to healthy foods), including children of Mexican-origin, energy-dense foods may also be more accessible, available, and affordable [2,15-17]. This may be especially true for Mexican-origin children in Texas border *colonias *who reside in food-insecure households in communities lacking access to nutritious food [2,3,18]. For children, residing in a food-insecure household can prevent them from achieving the nutrient intake needed for optimal development and health, as well as impede their academic performance [19-26].

Thus, it is critically important to understand the relationship between food insecurity and children's dietary intake among limited-resource Mexican-origin children. However, few studies have examined this association. Prior studies among Hispanics relied exclusively on parental reports of household food-supply adequacy and their child's diet and experiences. These studies revealed multiple associations between food insecurity and diet. Food-insecure children were less likely to meet recommended food-group guidelines [27], have greater intakes of fats, saturated fats, sweets, and fried foods [28], and lower fruit and vegetable intake at home [29]. Among 5^th ^grade students who reported dietary intake using three 24-hour dietary recalls and whose mothers reported food security, food-security status was not associated with dietary intake [30]. Only one study assessed dietary intake through child-reported dietary recalls, and none measured food security from the child's perspective.

Although mothers often spare their children from nutritional deprivation and report that children are more protected from household food insecurity [31,32], this experience is from the parent's perspective [33]. There has been a call for research to assess the relationship between food security and children's diet [34], yet little research has focused on child's perceptions or experiences of food insecurity and their association with dietary intake [35]. Current measures represent household food security status of the household or children within the household as a group, rather than the experiences of a particular individual within the household [36]. Children best report their own experiences [37]. Measurements of food security as reported by the child, which may be more sensitive of the food issues experienced by children, is essential for understanding the influence of food insecurity on the nutritional health of children [35]. Understanding the relationship between children's experience of food security and their dietary intake [30] is needed to comprehend the effect of food insecurity on children's nutrient intake [38]. The current study seeks to assess the relationship between children's experience of food insecurity and nutrient intake from food and beverages by (1) assessing food security status as reported by 50 Mexican-origin children (ages 6-11 years), (2) examining nutrient intakes from three 24 hour dietary recalls from each child, and (3) determining the relationship between food security status and nutrient intake.

## Methods

### Setting

The study was conducted in two large areas of *colonias *in Hidalgo County, located in the Lower Rio Grande Valley of Texas along the Mexico border. From prior work and in consultation with community partners [2], 10 census block groups (CBG) were identified in the western part of the county and 10 in the eastern portion of the county. In both areas, a majority of CBGs are considered to be highly deprived neighborhoods [2]. Highly deprived neighborhoods are those with overall high proportions of unemployed adults, households without telephone service, families receiving public assistance, households lacking complete kitchen facilities, households lacking complete plumbing facilities, adults with less than 10 years of education, or those living below the poverty threshold [2]. Forty *colonias *were spatially selected, with at least one *colonia *in each of the 20 CBGs.

### Study sample

The study sample consisted of 50 family dyads (mother and child 6-11 years), who were recruited for a cohort study by team *promotora*-researchers; 25 dyads were recruited from western area *colonias *and 25 from eastern area *colonias*. Letters of invitation were personally delivered by *promotora*-researchers, and eligibility was determined by the presence of one child (age 6-11 years) residing in the household. The study was explained to each prospective adult participant (e.g., inform about assessments, confidentiality, financial incentive, etc.), and the first of three in-home assessments was scheduled within two days. The mother provided consent for both members of the dyad to participate in the study, and the child provided assent for participation. All materials and protocols were approved by the Texas A&M University Institutional Review Board.

### Data collection

This analysis focuses on data collected March to June 2010 from all 50 children during three in-home visits: survey data and anthropometrics from the first visit and dietary recalls from all three visits. The survey included sections on demographics and food security and was interviewer-administered by *promotora*-researchers, who received training in collection of survey, anthropometric measures, and 24-hour dietary recalls. All materials were reviewed by community partners and were validated by local/area experts. A pilot test was conducted in *colonias *not selected from the study area and necessary modifications were made. *Promotora*-researchers received the equivalent of four full days of training on data collection and protection of participant confidentiality. All measures were translated into Spanish using translation-back translation method with the following steps: 1) translation of the original English into Spanish, ensuring that the English meaning is maintained; 2) back-translation into English by an independent translator who is blinded and is not familiar with either the Spanish or English version; 3) comparison of the two English versions; and 4) resolution of any discrepancies. Community partners and *promotora*-researchers verified translation accuracy and appropriateness to ensure semantic, conceptual, and normative equivalence. All survey and 24-hour dietary recall data were collected in Spanish, which was the language spoken in the homes of all participants.

### Measures

***Demographics ***included child's sex, age, school grade, and country of birth.

***Anthropometric ***measure of body mass index (BMI) was used to gain a general sense of body fatness. Weight was measured (to the nearest 0.1 kg) in the home with a portable, self-zeroing scale. Weight was measured twice, with the children wearing light clothing and no shoes. Standing height (to the nearest mm) was measured twice with a portable stadiometer. Using the mean of the two measures of weight and height, BMI was calculated as weight (kg)/[height (m)] ^2^. Appropriate Centers for Disease Control and Prevention (CDC) BMI-for-age-and-sex growth charts were used to classify each child's BMI status as underweight (< 5^th ^percentile), healthy weight (5^th ^percentile to < 85^th ^percentile), overweight (85^th ^percentile to < 95^th ^percentile), or obese (≥95th percentile) [39,40].

***Children's food security ***was assessed using the nine-item child food security measure developed by Connell and colleagues [33]. Pilot testing of the food security measure with a sample of children similar to participant children was performed to determine understandability and face validity. Participant children were asked by the *promotora*-researcher whether they experienced each of the nine items during the last three months (see Table 1). Response options included "a lot", "sometimes," or "never." Each item was constructed as a binary variable; yes (a lot or sometimes) vs. no (never). Iterative common factors analysis on the nine items identified one factor (eigenvalue = 3.2) that explained 79% of the shared variance; and internal reliability was good (Cronbach's α = 0.81). Affirmative responses to the nine items were summed into an ordinal children's food security score [34], which was used to categorize each child as having high food security (score = 0), marginal food security (score = 1), low food security (score = 2-4), or very low food security (score = 5-9).

**Table 1:** Children self-reported food security (*n *= 50)

***In the last 3 months***,	N (%)^1^
1. Did you worry that food at home would run out before your family got money to buy more?	25 (50)

2. Did the food that your family bought run out and your family did not have money to get more?	23 (46)

3. Were you not able to eat a variety of healthy foods at a meal because your family didn't have enough money?	20 (40)

4. Did your meals only include a few kinds of cheap foods because your family was running out of money to buy food?	27 (54)

5. Was the size of your meals cut because your family didn't have enough money for food?	17 (34)

6. Did you have to eat less because your family didn't have enough money to buy food?	16 (32)

7. Did you have to skip a meal because your family didn't have enough money for food?	15 (30)

8. Were you hungry but didn't eat because your family didn't have enough food?	4 (8)

9. Did you not eat for a whole day because your family didn't have enough money for food?	6 (12)

***Food Security Categories***	

High food security	9 (18)

Marginal food security	9 (18)

Low food security	18 (36)

Very low food security	14 (28)

^1^Affirmative response = combination of "a lot" and "sometimes"

#### Dietary intake

Three 24-hour (previous day) dietary recalls occurring on randomly selected, nonconsecutive days (one recall measured weekend intake and two measured weekday intake) were collected in the home from each participant child by the same interviewer (*promotora*-researcher). In most cases, the mother observed and assisted the children if necessary. Dietary intake training for the interviewers included review of all protocols and scripts, modeling of interviewing, practice interviews with children similar in age to the study participants, use of tools for portion-size estimation, quality control, and focus on children's reporting of food items. The first recall occurred during the first in-home visit, and the second and third recalls were collected in the home during the second and third visits (within two weeks of the first visit). Detailed information on food and beverage consumption, including description, brand name, location of preparation and consumption, and preparation method during the previous day was collected using standardized protocols following a modification of the multiple-pass interview technique of the Nutrition Data System for Research (NDS-R) [41]. Data were collected on hard copy in Spanish, modified from an approach previously used [42], and then entered into NDS-R 2009 in English [41]. Children were first asked to provide a quick list of generic food and beverage items consumed during the previous day based on short time intervals (e.g., before breakfast, at breakfast, between breakfast and lunch, and at dinner); prompts included food consumption occasions and locations. This was followed by a review of the quick list. During this pass, the interviewer probed for forgotten foods by asking about snacks and beverages (including water) and about the source of the food or beverage. The third pass provided food details such as the time and place of the eating occasion, food descriptions, brand name, ingredients and preparation, and portion size and quantity consumed. As a result of pilot testing, multiple approaches were used for estimation of portion size and included measurement of typically-used cups, glasses, bowls, and containers in the home, food and beverage models, geometric shapes (circles, rectangles, and wedges), and three-dimensional thickness aides. The fourth pass was a final and comprehensive review of the previous-day's intake. Nutrient calculations were performed using NDS-R 2009 software. Three-day mean nutrient intakes, with equal weighting for each of the three days (2 weekdays and 1 weekend) of dietary recall were calculated for each child for total energy (kcal), protein (g), dietary fiber (g), calcium (mg), vitamin D (mcg), potassium (mg), sodium (mg), Vitamin C (mg), percentage of calories from fat, percentage of calories from added sugars, and percentage of calories from saturated fat.

### Analysis

All analyses were performed using Stata Statistical Software: Release 11 (College Station, TX: StataCorp, 2009). Descriptive statistics were calculated for each child's baseline characteristics, BMI status, food security status, and nutrient intake. Wilcoxon Signed-Rank Test was used to compare weekend and weekday nutrient intake by level of food security. Non-parametric test for trend across ordered groups of food security was used to examine each nutrient. Separate multiple regression models with robust (White-corrected) Standard errors (SEs), were individually fitted for total energy (kcal), protein (g), dietary fiber (g), calcium (mg), vitamin D (mcg), potassium (mg), sodium (mg), Vitamin C (mg), percentage of calories from fat, percentage of calories from added sugars, and percentage of calories from saturated fat. All models included sex, age, country of birth, BMI status, and food security status as independent variables. These variables were selected based on their documented association with dietary intake.

## Results

Table 2 shows baseline characteristics for the 50 participant children. Sixteen children (32%) were born in Mexico and twenty-one (42%) were overweight or obese based on the Centers for Disease Control and Prevention BMI-for-age-and-sex growth charts [40]. Results from the nine-item children's food security measure are shown in Table 1. Thirty-two children (64%) reported low (*n *= 18) or very low food security (*n *= 14). Although BMI status was not significantly associated with food-security status, 48 percent of the sample was measured as being of healthy weight and reporting low or very low food security. Nutrient intake and dietary recommendations for the entire sample and nutrient intake by food-security status are shown in Table 3. Using the 2010 Institute of Medicine age- and sex-specific recommendations [43], 28% (*n *= 14) met the recommendations for calcium, none for potassium or vitamin D, 10% (*n *= 5) for dietary fiber, and 6% (*n *= 3) for sodium (data not shown). Although all children exceeded the recommendation for protein, as a percent of total calories, protein intake ranged from 11.8% to 22% (data not shown). Weekend intakes for calcium, vitamin D, potassium, and vitamin C were significantly lower than weekday consumption, and percentage of calories from fat, and combined percentage from fat and added sugar (data not shown) were significantly higher on weekends than weekdays. Children who were identified with low food security consumed significantly less calcium and vitamin D on weekends, compared with weekdays, and very low food-security children consumed a greater percentage of calories from fat on weekends than weekdays. Three-day average intake for total energy, protein, percentage of calories from added sugar, and percentage of calories from saturated fat demonstrated a significant and positive trend (indicating greater intake) with reduced food-security status. The same positive trend was observed for weekend intake of total energy, and for weekday intake of percentage of calories from added sugars and saturated fat.

**Table 2:** Baseline characteristics of Mexican-origin children (*n *= 50)

	Mean ± SD (Median)	N (%)
Sex		

Female		31 (62)

Age, y	9.1 ± 1.3 (9.2)	

Country of birth		

Mexico		16 (32)

United States		34 (68)

Weight status^a^		

Underweight		5 (10)

Healthy weight		24 (48)

Overweight		9 (18)

Obese		12 (24)

^a ^Based on BMI-for-age-and-sex growth charts. Underweight = < 5th percentile; healthy weight = 5th percentile to < 85th percentile; overweight = 85th percentile to < 95th percentile; and obese = ≥ 95th percentile

**Table 3:** Nutrient intake (3-day average, weekday average, and weekend) overall and by food security statusa

	Dietary Recommendations		Total	Food	Marginal food secure	Low food security	Very low food security
			Sample	secure			
	6-8 y	9-11 y	(*N *= 50)	(*N *= 9)	(*N *= 9)	(*N *= 18)	(*N *= 14)
Total energy (kcal)	1400-1600	1600-2200					

3-day average^c2 ^			2034.9	1766.9	2049.2	1994.2	2250.2

			(372.8)	(354.5)	(287.0)	(340.7)	(376.6)

Weekday			2008.1	1757.4	2081.7	2020.7	2105.8

			(375.8)	(313.9)	(318.0)	(363.4)	(423.0)

Weekend day^c2^			2097.5	1785.9	2023.0	1947.0	2539.1

			(717.6)	(761.6)	(687.5)	(611.9)	(700.9)

Protein (g)	19	34					

3-day average^c1^			82.9	73.3	78.4	84.8	89.5

			(19.7)	(17.9)	(14.1)	(20.2)	(22.0)

Weekday			81.1	70.7	78.8	86.7	82.1

			(19.9)	(18.0)	(11.8)	(21.7)	(21.9)

Weekend day			86.4	78.6	76.5	81.6	104.1

			(36.7)	(36.3)	(30.2)	(35.8)	(39.3)

Dietary fiber (g)	17-20	22-25					

3-day average			15.2	15.7	14.3	16.2	14.1

			(5.0)	(3.3)	(4.2)	(5.3)	(6.2)

Weekday			15.5	16.6	16.2	16.5	13.1

			(5.3)	(5.1)	(5.6)	(5.8)	(4.3)

Weekend day			14.6	13.8	10.8	15.6	16.1

			(9.8)	(6.9)	(4.6)^b1^	(7.1)	(15.4)

Calcium (mg)	1000	1300					

3-day average			993.4	820.4	1077.5	951.6	1104.3 (387.6)

			(300.5)	(266.4)	(245.4)	(228.1)	

Weekday			1056.3	918.2	1131.5	1095.7	1046.2

			(299.6)	(360.2)	(149.1)	(321.3)	(300.8)

Weekend day			898.2	624.7	999.7	733.6	1220.6

			(566.2)^b2^	(355.8)	(609.7)	(381.5)^b2^	(709.3)

Vitamin D (mcg)	15	15					

3-day average			6.9	5.4	7.6	6.2	8.4

			(2.9)	(2.9)	(1.9)	(2.5)	(3.4)

Weekday			7.5	6.3	7.9	7.2	8.6

			(2.7)	(3.1)	(2.6)	(2.2)	(3.0)

Weekend day			5.8	3.6	6.8	4.6	8.0

			(4.4)^b3^	(3.5)^b1^	(2.1)	(4.3)^b2^	(5.1)

Potassium (mg)	3800	4500					

3-day average			2530.1	2499.1	2489.6	2457.6	2669.4

			(554.4)	(482.4)	(451.5)	(530.4)	(701.4)

Weekday			2640.3	2649.6	2692.4	2650.3	2588.0

			(602.8)	(573.2)	(466.5)	(695.9)	(628.9)

Weekend day			2332.9	2197.9	2052.4	2152.2	2832.3

			(955.2)^b1^	(976.7)	(716.3)	(782.3)	(1164.0)

Sodium (mg)	< 1900	< 2200					

3-day average			3450.7	3083.0	3165.0	3687.8	3565.9

			(913.2)	(750.9)	(330.6)	(1050.7)	(1021.4)

Weekday			3372.1	3100.0	3180.7	3698.0	3251.0

			(994.8)	(881.8)	(357.7)	(1227.5)	(981.2)

Weekend day			3553.5	3049.0	3042.9	3561.5	4195.7

			(1695.0)	(1074)	(1198.7)	(1934.2)	(1881.8)

Vitamin C (mg)	25	45					

3-day average			101.1	114.2	92.7	87.8	113.7

			(52.7)	(64.6)	(55.9)	(36.8)	(59.4)

Weekday			110.5	124.7	105.1	93.8	122.6

			(63.8)	(72.2)	(69.2)	(57.4)	(64.2)

Weekend day			81.4	93.3	62.2	78.9	95.8

			(70.4)^b2^	(98.6)	(56.8)	(52.8)	(82.0)

Fat (percent of calories)	25-35	25-35					

3-day average			34.0	32.2	33.0	35.3	34.2

			(4.1)	(4.4)	(3.8)	(4.5)	(3.2)

Weekday			32.5	30.3	32.9	33.4	32.7

			(4.2)	(3.2)	(5.3)	(4.4)	(3.7)

Weekend day			36.8	35.9	33.6	38.7	37.1

			(7.8)^b3^	(10.1)	(4.0)	(9.7)	(4.9)^b1^

Added sugars (percent of calories)							

3-day average^c1^			15.0	11.8	16.5	13.0	18.7

			(5.5)	(2.8)	(5.3)	(4.5)	(6.0)

Weekday^c1^			14.8	11.1	15.3	13.5	18.7

			(6.3)	(4.1)	(5.2)	(6.0)	(7.0)

Weekend day			15.5	13.5	20.0	12.1	18.3

			(7.7)	(4.7)	(9.0)	(6.7)	(7.9)

Saturated fat (percent of calories)	< 10	< 10					

3-day average			12.6	11.4	12.8	12.8	12.8

			(2.0)	(2.0)	(1.4)	(2.2)	(1.9)

Weekday			12.3	11.4	13.1	12.5	12.0

			(2.2)	(1.7)	(2.3)	(2.4)	(2.2)

Weekend day			13.2	11.4	13.1	13.4	14.2

			(4.2)	(3.8)	(3.8)	(4.7)	(3.8)

Dietary Recommendations for children from USDA/ARS Children's Nutrition Research Center at Baylor College of Medicine, available at http://www.bcm.edu/cnrc/consumer/archives/percentDV.htm

^a ^Nutrient intake reported as mean (SD)

^b ^Comparison of weekday and weekend day nutrient intake by level of food security, using the Wilcoxon Signed-Rank Test

^c ^Test for trend across ordered groups of food security

Level of statistical significance: ^1^*p *< 0.05 ^2^*p *< 0.01 ^3^*p *< 0.001

The multiple regression results, presented in Table 4, show the association of children's food-security status, sex, age, BMI status, and country of birth to nutrient-specific intakes. Very low food security was associated with greater intakes of total energy, calcium, and percentage of calories from added sugar. In data not shown in Table 4, marginal (β = 4.8, SE = 2.2, *p *= 0.032), low (β = 4.4, SE = 1.9, *p *= 0.028), and very low (β = 8.4, SE = 2.0, *p *< 0.001) food security were associated with increased intake as a percentage of calories from combined fat and added sugar. In addition, increased age was associated with lower intakes of calcium and vitamin D; being born in Mexico with greater intake of sodium; and being female with lower calcium intake. BMI status was not associated with any of the nutrients.

**Table 4:** Children's food security and demographic correlates of children's nutrient intakes from multiple regression models1

	Energy	Protein	Fiber	Calcium	Vitamin D	Potassium	Sodium	Vitamin C	Fat	Added Sugar
	**(kcal)**	**(g)**	**(g)**	**(mg)**	**(mcg)**	**(mg)**	**(mg)**	**(mg)**	**(% kcal)**	(% kcal)

Food security										

Marginal	238.06 (142.06)	2.34 (8.16)	-0.65 (2.21)	270.57 (109.28)**	1.93 (1.24)	-96.78 (235.54)	-62.01 (288.16)	-16.45 (25.06)	0.16 (2.22)	4.15 (2.14)

Low	166.71 (140.92)	8.54 (7.39)	0.16 (2.02)	91.00 (102.56)	0.60 (1.09)	-109.31 (190.62)	377.84 (371.94)	-30.17 (21.19)	2.25 (1.77)	1.65 (1.48)

Very low	377.16 (169.91)*	10.54 (9.32)	-0.94 (2.30)	187.29 (139.25)*	1.71 (1.40)	22.11 (275.27)	278.73 (365.86)	-3.68 (25.64)	0.97 (1.80)	6.82 (2.05)***

Female	-118.83 (124.52)	-5.51 (6.87)	-1.20 (1.89)	-189.29 (84.62)*	-0.86 (0.82)	-130.99 (194.59)	-124.02 (362.36)	0.66 (19.27)	-1.44 (1.40)	2.31 (1.56)

Age	-48.27 (54.08)	-2.24 (2.58)	0.89 (0.69)	-83.56 (29.02)**	-1.18 (0.28)***	-67.15 (76.77)	-30.30 (115.29)	-6.74 (6.44)	-0.35 (0.45)	0.04 (0.54)

BMI status										

Overweight	-60.29 (150.37)	-4.65 (8.32)	0.65 (2.16)	50.17 (79.68)	0.42 (0.77)	-183.03 (210.69)	7.43 (440.01)	-3.19 (21.13)	-0.85 (1.77)	0.12 (1.62)

Obese	22.25 (121.58)	2.09 (7.37)	0.35 (1.44)	50.11 (109.81)	1.05 (1.04)	-11.24 (202.18)	-81.29 (300.96)	-21.66 (17.85)	-1.87 (1.64)	2.02 (1.68)

Country of birth										

Mexico	78.80 (118.59)	3.73 (6.49)	1.66 (2.13)	-53.06 (80.62)	-0.85 (0.59)	43.00 (211.25)	689.84 (337.07)*	-10.73 (20.01)	0.76 (1.44)	0.23 (1.73)

*R^2^*	0.271	0.156	0.120	0.353	0.478	0.078	0.220	0.118	0.145	0.290

^1 ^Coefficients are reported; robust SEs are in parenthesis and are corrected with the White-Huber correction. There were 50 observations. Reference categories (variables) omitted to prevent perfect collinearity: food security (food secure), female (male), BMI status (Normal/Underweight), and country of birth (U.S.). Age was entered as a continuous variable

* *p *< 0.05 ** *p *< 0.01 *** *p *< 0.001

## Discussion

Previous work has recognized the relationship between food security and children's diets [34,35,37], but this is apparently the first study to assess the relationship between food security and children's nutrient intakes in Mexican-origin children, based on children's reports of both their experiences of food insecurity and dietary intake. The national prevalence of household food insecurity is greater among Hispanic households in the U.S [7] and substantially greater among Mexican-origin households in *colonias *[3]. Findings from this study expand our understanding of the experience of food insecurity by school-age, Mexican-origin children and the association of food-security status with nutrient intakes.

Results present additional evidence that food insecurity is more prevalent among Mexican-origin children in Texas border *colonias *than previous estimates suggested. For instance, national data from 2009 indicated that 18.7% of Hispanic households, regardless of race or country of origin, had food-insecure children [7]. A community-based nutrition assessment of 610 Mexican-origin adults in Texas border *colonias *reported that 49% of all households and 61.8% of households with children were classified as child food insecure [3]. In the current sample, 28% of children reported very low food security and 64% reported low or very low food security. At least one-third of children reported having to skip a meal, go hungry, or not eat for a whole day because of limited or no food resources in the home, which supports and is supported by the community assessment. Children's total energy and nutrient intakes in this study were similar to or greater than previously reported among Mexican-American children (6-11 years) [44]. In addition, the present study showed that decreasing food-security status was associated with increased intake of total calories and percentage of calories from fat and added sugars, which confirms the work of Rosas and colleagues [28], and is in contrast to the work of Matheson and colleagues, which reported a not-significant relationship between household food security and children's dietary intake [30].

This paper not only emphasizes the alarming rates of food insecurity for this Hispanic subgroup, but describes the associations for food insecurity and diet among this sample of Mexican-origin children. Such findings have implications at a regional and national level, as the Mexican-origin population continues to grow along the border and in new destination communities [8,45]. Immigrants from Latin America have provided the largest percent of foreign-born population since 1990: 44.3% in 1990, 51.7% in 2000, and 53.6% in 2007 [46]. People of Mexican-origin represent approximately 64% of both the native and foreign-born Hispanic population [46]. Of the 29.2 million Mexican Hispanics, 40 percent were foreign born. The percentage of all children living in the United States with at least one foreign-born parent increased from 15 percent in 1994 to 23 percent in 2010 [47]. In 2010, 33 percent of foreign-born children with foreign-born parents and 26 percent of native children with foreign-born parents lived below the poverty line [47]. Data presented here may foreshadow higher rates of nutrition-related health conditions, such as obesity, type 2 diabetes, and cardiovascular disease among Mexican-origin children. Child-reported food insecurity situations could serve as a screen for nutrition problems in children. Further, the National School Lunch and School Breakfast Programs, which play a major beneficial role in children's weekday intakes, may not be enough to keep pace with the nutritional needs of low and very low food secure Mexican-origin children.

The present study has several particular strengths. First, this is a study of hard-to-reach Mexican-origin children in border *colonias*. This population is of increasing national importance because such *colonias *can be considered an archetype for the new-destination Mexican immigrant communities that are now found in great numbers throughout the U.S. Second, to our knowledge, this is the first study that uses children's report of their food-insecurity experiences in the past three months to describe food-security status, which is preferable and reduces the cognitive burden placed on respondents by the conventional twelve-month time period [7]. As such, this study builds on the work of Connell and colleagues and Fram and colleagues [33,35,37], that identified the importance of the child's perspective in understanding food insecurity and its consequences [35,37]. Third, usual dietary intake was determined by three 24-hour dietary recalls that included weekday and weekend intakes. Each recall was conducted face-to-face in the home, multiple strategies were used to estimate portion size, and a modified multiple-pass method was used to capture home and away-from-home (e.g., school) food intake. Young children can provide information on their diet as accurately, or more accurately, than their parents, especially for food eaten outside the home [48-51].

There are several limitations to this study that warrant mention. Data were collected during one season of the year, which limits our ability to describe seasonal variation in dietary intake or food-security status or to make causal inferences. This could have important implications for times of year when children are unable to participate in school nutrition programs, such as during the summer or holidays. Although the three-month time frame was much better than asking about food security experiences in the last 12 months, this study did not collect data on frequency or duration of food insecurity situations. This limits our ability to differentiate between acute and chronic food insecurity. An additional limitation is an absence of data on food coping strategies employed by children to help manage food resources [37]. Finally, the study sample is small and is limited to two areas of *colonias *in the Texas border region, which limits our ability to generalize these results. Future work should focus on expanding our understanding of seasonal variation in the frequency and duration of children's experiences of food insecurity.

## Conclusions

Despite these limitations, the results of this study further the knowledge of children's experiences of low and very low food security and the association of food security status with children's dietary intake. The Mexican-origin population is rapidly expanding throughout the United States; record numbers of individuals and families are experiencing food insecurity, and for children living in rural or underserved areas such as the *colonias*, food insecurity may be an ongoing reality. The prevalence of low and very low food security in this border area is alarming, despite the participation of all study participants in the School Breakfast and National School Lunch Programs. The high prevalence of low and very low food security among these children is especially troubling given the importance of good nutrition on optimal growth, function, and health [19,20]. Young children of Mexican immigrant families have a greater risk for hunger and household food insecurity [8], and are less likely to meet dietary recommendations than other children [27]. In this sample of Mexican-origin children, not only did most of the children not meet dietary recommendations in key nutrients, but children with very low food security consumed higher levels of energy, fat, and added sugars. The results also indicate the importance of further examining the frequency and duration of low and very low food security in children. Enhanced research efforts are needed that will lead to better understanding of coping strategies and the use of federal and community food and nutrition assistance programs for reducing food insecurity. Clearly, systematic and sustained action on multiple levels that integrates multi-sector partnerships and networks is needed for culturally-tailored health promotion and policy efforts to reduce child food insecurity.

## Competing interests

The authors declare that they have no competing interests.

## Authors' contributions

JRS designed the study, and worked on the development of the instrument and the protocol for collection of data. JRS, CN, CMJ, and WRD wrote the first draft of the paper. JRS, CN, CMJ, and WRD read and approved the final manuscript.

## Authors' information

JRS is Professor of Social and Behavioral Health and Director of the Program for Research in Nutrition and Health Disparities; CN is a Graduate Research Assistant; CMJ was a Research Associate; and WRD is Assistant Professor of Social and Behavioral Health.

## Pre-publication history

The pre-publication history for this paper can be accessed here:

http://www.biomedcentral.com/1471-2431/12/16/prepub
